# “Students in public and private schools—which are at higher risk of drug use?”: a survey from Iran

**DOI:** 10.1186/s13011-020-00330-1

**Published:** 2020-11-23

**Authors:** Ali Bahramnejad, Abedin Iranpour, Nouzar Nakhaee

**Affiliations:** 1 Neuroscience Research Center, Institute of Neuropharmacology, Kerman University of Medical Sciences, Kerman, Iran; 2 Research Center for Social Determinants of Health, Institute for Futures Studies in Health, Kerman University of Medical Sciences, Kerman, Iran; 3 Health Services Management Research Center, Institute for Futures Studies in Health, Kerman University of Medical Sciences, Kerman, Iran

**Keywords:** Students, Teenagers, Substance use, Socioeconomic class

## Abstract

**Background:**

Recent evidence from Western countries suggests that private school students are more prone to drug use. Such an evidence is lacking in Muslim countries. The aim of this study was to examine whether the risk of drug use is higher in private schools than public schools.

**Methods:**

This cross sectional study was conducted on 650 tenth grade students of Kerman city, the center of largest province of Iran using cluster sampling. Well-validated questionnaires regarding current, lifetime substance use, and perceived use by classmates were utilized. Substances included in the questionnaire were waterpipe, cigarette, alcohol, marijuana, opium, methamphetamine, and Naas. Drug Use Tendency Scale was used to measure the attitudes of students towards drug use.

**Results:**

The response rate was 93.7%. More than 82% of sample were public school students (*n* = 504). Current use of cigarette and marijuana was higher in private schools (12.2 and 3.0%, respectively) than public schools (4.4 and 0.5%, respectively) (*P* < 0.05). Perceived prevalence of cigarette smoking by classmates was higher among private school students.

**Conclusion:**

Despite the popular belief that private schools are better than public schools regarding the risk of substance use, students who attend private schools may be at a higher risk of turning to some drugs comparing to public schools in Iran.

## Background

Drug use is a major threat to public health worldwide. According to the United Nations Office on Drugs and Crime (UNODC) World Drug Report 2018, prevalence of drug use among older population remains lower than among young individuals [1]. The most susceptible group to initiation of drug use are adolescents. A variety of biological, psychological, and environmental factors contribute to the vulnerability of this age group, such as maturing brain, peer influence and decreased harm avoidance [2]. Worldwide tobacco, alcohol, and marijuana are the three most commonly used drugs by adolescents [1]. The pattern of drug use amongst high school students varies from country to country. According to the Youth Risk Behavior Surveillance System (YRBSS) 8.8% of US high school students are current smokers and 29.8% current alcohol users [3]. The 2011 European School Survey Project on Alcohol and Other Drugs (ESPAD) report showed that 28% of the students in the 36 participating countries were current cigarette smokers and 57% of them have used alcohol the 30 days before the survey (i.e., current alcohol use) [4]. The prevalence of current cigarette and current alcohol use among Iranian high school students was 5.6 and 9.9%, respectively [5]. Prevalence of regular waterpipe smoking among Iranian adolescents was highest in the world according to a recent review [6].

Studies emphasize the role of the classmates and school environment in initiating drug use by high school students [7]. While no specific influential factor alone is sufficient to lead to drug use, one of the most important environmental factors that may exacerbate or reduce the likelihood of drug use amongst youth is the school culture [8]. School culture consists of shared values, behaviors and norms [8]. In many countries such as England, France, Finland [9], and China [10] private schools are more likely to serve students from high socioeconomic class. In Iran owing to the high cost of private education such a pattern is seen too. In Iran as in many other countries, it is widely accepted that private schools are superior to public schools. Parents believe that sending their child to private schools might better prepare them for the future [11]. They think that pupils who go to private schools are less likely to suffer from drug use since privileged students are better educated about anti-drug life skills programs and the private schools’ environment is less risky. There are very few studies comparing private and public schools regarding the prevalence of drug use and other risky behaviors [12]. Moreover, the paucity of studies that have assessed the issue are from Western countries [13, 14]. A research conducted on upper middle-class youth in affluent communities of United States (US) revealed that those who go to private schools are at higher likelihood of drug and alcohol use [13]. Another US study pointed that attending schools with a high proportion of affluent schoolmates increased the risk of drug use [14].

We conducted this survey to answer the popular question, “Are private schools better than public schools regarding vulnerability to drug use?”. We assessed three domains to compare the school climate regarding likelihood of drug use between public and private schools in Iran; frequency of drug use by students, the drug use tendency of students, and perception of students regarding prevalence of drug use in classmates (i.e., perceived prevalence). To the best of our knowledge, this is the first study, which compares the risk of drug use between public and private schools in a non-Western culture.

## Methods

### Setting

This cross sectional study was conducted on the 10th grade high school students of Kerman city, the center of Kerman province, Iran. Twelve schools were selected using cluster sampling method. The sampling was proportional to size. There are 28,541 students in the city’s high schools, 14.8% of whom are in private schools. The ratios in public and private schools were proportionate to the population of students in the public and private students. In each school, all 10th grade students were invited to take part in the study. Grade 10 students were selected because it has been shown that this grade is a transition-linked turning point between middle school and high school and provides opportunities for new risky behaviors [15]. To ensure confidentiality of the responses, a sealed ballot box was placed at the center of classroom [16]. The seats were spaced far apart enough to warrant privacy. Except for age, other demographic questions were not included in the questionnaire to ensure students that their responses would not be identifiable [16]. Since in Iran all schools are single-sex we did not ask them about their gender.

### Measurements

We used three questionnaires to collect the data. The first tool was the Drug Use Tendency Scale which consists of twelve items using five point Likert scale from 0 to 4 (strongly disagree to strongly agree). A higher score denoted more tendency toward drug use [17]. It was part of a two-factor questionnaire originally developed to measure adolescent tendency to engage in high-risk behaviors. The first 12 items of this questionnaire were specifically devoted to measure adolescents’tendency towards drug use [17]. Sample items were such as “I go to parties where drugs are consumed” or “Smoking a hookah relaxes me”. The second part measured current (past 30 days) and ever (lifetime) drug use among students [5]. We included the seven most prevalent drugs among Iranian students (i.e., waterpipe, alcohol, cigarette, marijuana, opium, methamphetamine, and Naas) [5]. Naas or Naswar as a type of smokeless tobacco product is a green powder tobacco stuffed in the floor of the mouth or between the oral mucosa and gingival cavity [18]. The third part queried about perceived use of the above-mentioned drugs by classmates [19]. To ensure untraceability of respondents minimum demographic items (i.e., age, sex, and type of school) were included in the survey.

### Ethical considerations

Ethics Committee approved the research protocol. All questionnaires were anonymous and unlinked. The respondents were assured about the privacy of data. Informed consent was obtained from both students and their parents.

### Statistical analysis

Taking into consideration the nested nature of the data, cluster adjusted prevalence estimates were calculated and compared between the two groups using chi square test. Stata version 16.0 was used for statistical analysis. All analyses were performed accounting for the clustered and weighted survey design using the svy commands (with *pweights* specified) based on a first-order Taylor series approximation in Stata [20].

## Results

From 650 students invited to participate in the study 609 subjects completed the questionnaires (i.e., a response rate of 93.7%).

Boys constituted 58.3% of participants (*n* = 355). More than two-thirds of them were 16 years old and the remaining were 15 years old (23%). More than 82% of sample were from public schools (*n* = 504). The drug use tendency score in public schools and private schools was (10.4 ± 9.4) and (12.1 ± 9.9), respectively (*P* = 0.090).

Regarding lifetime substance use in both public and private schools, the most common practices were smoking waterpipe (45.6%) and using alcohol (26.3%). Prevalence of current and ever use of various drugs according to school type is shown in Tables 1 and 2.

**Table 1:** Frequency (%) of ever drug use among 10th grade high school students based on school type (*n* = 609)^a^

Drug	Total	Public	Private	Pearson’ s Chi-Square	***P***-value
Cigarette	16.6%	14.7%	27.7%	12.19	0.01
Waterpipe	45.6%	46.1%	43.0%	0.15	0.71
Alcohol	26.3%	24.4%	37.2%	2.41	0.15
Opium	2.5%	2.8%	0.7%	2.09	0.18
Naas	1.1%	1.3%	0.0%	0.28	0.61
Marijuana	2.4%	2.0%	4.5%	1.00	0.34
Methamphetamine	0.3%	0.2%	0.7%	1.27	0.28

^a^ Percentages were adjusted for school cluster sampling

**Table 2:** Frequency (%) of current drug use among 10th grade high school students based on school type (*n* = 609)^a^

Drug	Total	Public	Private	Pearson’ s Chi-Square	***P***-value
Cigarette	5.5%	4.4%	12.2%	7.83	0.02
Waterpipe	19.3%	19.9%	15.7%	0.80	0.39
Alcohol	11.4%	11.8%	8.8%	0.30	0.59
Opium	0.8%	0.8%	0.7%	0.01	0.94
Naas	0.3%	0.4%	0.0%	0.12	0.73
Marijuana	0.9%	0.5%	3.0%	3.68	0.04
Methamphetamine	0.3%	0.2%	0.7%	1.27	0.28

^a^ Percentages were adjusted for school cluster sampling

Perceived prevalence of drug use by classmates is shown in Table 3. Waterpipe smoking and cigarette smoking were the two most prevalent drugs used by classmates according to the report of students in both public and private schools. Alcohol and methamphetamine use were reported less than other drugs.

**Table 3:** Perceived prevalence of drug use among 10th grade high school students based on school type (*n* = 609)^a^

Drug	Total	Public	Private	Pearson’ s Chi-Square	***P***-value
Cigarette	66.1%	63.7%	80.5%	3.96	0.04
Waterpipe	86.4%	86.8%	84.1%	0.22	0.65
Alcohol	8.2%	7.8%	10.7%	1.05	0.33
Opium	21.1%	22.9%	10.3%	2.03	0.18
Naas	28.4%	30.2%	18.4%	0.76	0.40
Marijuana	34.7%	33.6%	41.1%	0.38	0.55
Methamphetamine	10.1%	10.1%	10.0%	0.00	0.98

^a^ Percentages were adjusted for school cluster sampling

## Discussion

The role of family and peers on the risk of youth substance use have been widely highlighted in the literature, but in comparison, the role of school has not received as much attention. Results from this study suggest that the current (past 30 day) prevalence of cigarette and marijuana is higher in private schools than public schools. Ever use of cigarette was significantly more prevalent in private schools than public schools.

As a whole, waterpipe, alcohol, and cigarette were the three most frequently used drug during the past 30 days by students. In urban schools of the United States (US) alcohol (17.4%), marijuana (12.3%) and smoking tobacco (8.2%) were the three most frequently drugs in the past 30-day among the 10th Grade students [21]. According to ESPAD report past 30-day use of cigarette, alcohol, and marijuana by European students was 28, 57, and 7%, respectively [4]. Comparing to US and European high school students it seems that the prevalence of past month use of the abovementioned drugs is lower in our sample except for cigarette smoking in private schools.

Of the seven drugs studied, two drugs (i.e., cigarette and marijuana) showed higher past month use and cigarette showed higher lifetime use by students of private schools comparing to students of public schools. No drugs showed a higher prevalence in public schools than private schools. Luthar and Barkin showed higher rates of drinking to the point of intoxication among wealthier US students [22]. Other relevant studies has shown that affluent 10th-graders reported higher use of alcohol, marijuana and cigarettes than their inner-city counterparts [23]. An US nationally-representative study clarified elevated rates of drug use among affluent youth compared to national norms [14], as well as another US study which revealed that students with college-educated parents and those from high income families were more prone to binge drinking and marijuana use [24] . Similar findings has been shown among Brazilian high school students [25]. A survey among 15–16 year old UK students found a higher risk of alcohol consumption by those students who attended a school in the least deprived regional quintile and those who had more spending money per week [26]. Meanwhile, our study revealed that private school students scored similar on drug use tendency comparing public school students.

The Perceived prevalence of drug use by classmates showed roughly a similar trend. Perceived prevalence is an indicator of descriptive norm, which confers to a person’s perception of how widespread a behavior occurs by his/her referent others [27]. The higher the perceived prevalence of drug use by classmates, the higher the likelihood of engagement of high school students in drug use [28]. Overall, it seems that the high school students overestimated the prevalence of all drugs, which is in line with relevant studies [29].

The question we have to answer is why students of private high schools showed higher prevalence of some drugs and why they regarded cigarette use more normatively comparing to students of public schools? Studies conducted to compare risk of substance use between economically advantaged youth and teenagers in the lower socioeconomic classes pointed to several possible reasons. First, affordability and accessibility of drugs is a well-established risk factor for drug use in adolescents [30]. Second, parents from high-income families may have more laissez-faire attitudes toward alcohol and drug use [13]. Third, considering negative correlation between religiosity and socioeconomic status, which is ubiquitous among various religions including Islam [31], the protective role of Islamic beliefs against alcohol use would be less prominent among affluent students than more deprived students. Fourth, with the prevailing culture of individualism, which is more prominent among affluent families the youth from such families “can experience as much isolation from parents as do those at the lowest extreme” [23]. Fifth, parents with youth in private schools has higher expectations for studying hard [11]. In such a stressful atmosphere, the likelihood of engagement in drug use is higher than normal situations [14].

Parents enroll their children in private schools, hoping to study in a healthy environment. This study showed that there is no guarantee against drug use in private school space. Therefore, “it is simplistic to think of “good” schools and “bad” schools in terms of drug use” [32]. In the New Millennium, affluent youths are identified as an emerging at-risk group for substance use [33]. One limitation of our study is that we did not ask the students to identify their socioeconomic status and we did not included questions examining the role of parents and friends in shaping risk of drug use. So we were not able to draw a big picture regarding comparison between private schools and public schools.

## Conclusion

Putting in mind that the “role of family and peers” and the role of public/private schools are inextricably bound, this study showed that in Iranian community students in private schools are at higher risk for use of cigarette and marijuana. Parents need to get rid of the misconception that private schools protect youth from drug use. They should focus on their own role, instead of relying on schools to tackle the problem of drug use by teens.

## Data Availability

The data supporting the findings are available from the corresponding author on reasonable request.

## Abbreviations

EC: Ethics Committee
ESPAD: European School Survey Project on Alcohol and Other Drugs
UNODC: United Nations Office on Drugs and Crime
US: United States
YRBSS: Youth Risk Behavior Surveillance System

## References

[1] United Nations Office on Drugs and Crime (UNODC) (2018). World Drug Report.

[2] Gray KM, Squeglia LM (2018). Research review: what have we learned about adolescent substance use?. J Child Psychol Psychiatry.

[3] Kann L, McManus T, Harris WA, Shanklin SL, Flint KH, Queen B, Lowry R, Chyen D, Whittle L, Thornton J, Lim C (2018). Youth risk behavior surveillance—United States, 2017. MMWR Surveill Summ.

[4] Hibell B, Guttormsson U, Ahlström S, Balakireva O, Bjarnason T, Kokkevi A, Kraus L (2012). The 2011 ESPAD report. Substance use among students in.

[5] Bahramnejad A, Iranpour A, Nakhaee N. Gender-based differences in risk-taking behaviors among high school students in Southeast Iran. Int J Adolesc Med Health, 2020. 1 (ahead-of-print). https://www.degruyter.com/view/journals/ijamh/ahead-of-print/article-10.1515-ijamh-2019-0205/article-10.1515-ijamh-2019-0205.xml. 10.1515/ijamh-2019-0205 32549159

[6] Jawad M, Charide R, Waziry R, Darzi A, Ballout RA, Akl EA (2018). The prevalence and trends of waterpipe tobacco smoking: A systematic review. Plos One.

[7] Nakhaee N, Ziaaddini H, Karimzadeh A (2009). Epidemiologic study on drug abuse among first and second grade high school students in Kerman. Addict Health.

[8] Bisset S, Markham WA, Aveyard P. School culture as an influencing factor on youth substance use. J Epidemiol Community Health. 2007;61(6):485–90. 10.1136/jech.2006.048157 PMC2465725 17496256

[9] Dronkers J, Avram S (2010). Social class dimensions in the selection of a private school: a cross-national analysis using PISA. Educ Res Eval.

[10] Xuan X, Xue Y, Zhang C, Luo Y, Jiang W, Qi M, Wang Y (2019). Relationship among school socioeconomic status, teacher-student relationship, and middle school students’ academic achievement in China: using the multilevel mediation model. PLoS One.

[11] Sakellariou C (2017). Private or public school advantage? Evidence from 40 countries using PISA 2012-mathematics. Appl Econ.

[12] Pearman SN, Valois RF, Thatcher WG, Drane JW (2001). Physical activity behaviors of adolescents in public and private high schools. Am J Health Behav.

[13] Luthar SS, Small PJ, Ciciolla L (2018). Adolescents from upper middle class communities: substance misuse and addiction across early adulthood. Dev Psychopathol.

[14] Coley RL, Sims J, Dearing E, Spielvogel B. Locating economic risks for adolescent mental and behavioral health: poverty and affluence in families, neighborhoods, and schools. Child Dev. 2018;89(2):360–9. 10.1111/cdev.12771 PMC5573665 28245340

[15] Graber JA, Brooks-Gunn J (1996). Transitions and turning points: navigating the passage from childhood through adolescence. Dev Psychol.

[16] Tourangeau R, Yan T (2007). Sensitive questions in surveys. Psychol Bull.

[17] Bahramnejad A, Iranpour A, Karbakhsh M, Nakhaee N (2017). Development of risk-taking tendency tool for high school students. Addict Health..

[18] Saeed M, Muhammad N, Khan SA, Gul F, Khuda F, Humayun M, Khan H (2012). Assessment of potential toxicity of a smokeless tobacco product (naswar) available on the Pakistani market. Tob Control.

[19] Bahramnejad A, Iranpour A, Nakhaee N (2019). Is inclusion of a dummy drug necessary for estimating perceived prevalence of substance use by classmates?. Int J High Risk Behav Addict.

[20] Bell BA, Onwuegbuzie AJ, Ferron JM, Jiao QG, Hibbard ST, Kromrey JD (2012). Use of design effects and sample weights in complex health survey data: a review of published articles using data from 3 commonly used adolescent health surveys. Am J Public Health.

[21] Warren JC, Smalley KB, Barefoot KN (2017). Recent alcohol, tobacco, and substance use variations between rural and urban middle and high school students. J Child Adolesc Subst Abuse.

[22] Luthar SS, Barkin SH (2012). Are affluent youth truly “at risk”? Vulnerability and resilience across three diverse samples. Dev Psychopathol.

[23] Luthar SS, Sexton CC (2004). The high price of affluence. Advances in child development and behavior.

[24] Humensky JL (2010). Are adolescents with high socioeconomic status more likely to engage in alcohol and illicit drug use in early adulthood?. Subst Abuse Treat Prev Policy.

[25] Locatelli D, Sanchez Z, Opaleye E, Carlini C, Noto A (2012). Socioeconomic influences on alcohol use patterns among private school students in São Paulo. Braz J Psychiatry.

[26] Bellis MA, Hughes K, Morleo M, Tocque K, Hughes S, Allen T, Harrison D, Fe-Rodriguez E (2007). Predictors of risky alcohol consumption in schoolchildren and their implications for preventing alcohol-related harm. Subst Abuse Treat Prev Policy.

[27] Rimal RN, Real K (2003). Understanding the influence of perceived norms on behaviors. Commun Theory.

[28] Bertholet N, Faouzi M, Studer J, Daeppen JB, Gmel G (2013). Perception of tobacco, cannabis, and alcohol use of others is associated with one’s own use. Addict Sci Clin Pract.

[29] Debnam KJ, Saha S, Bradshaw CP (2018). Synthetic and other drug use among high school students: the role of perceived prevalence, access, and harms. Subst Use Misuse.

[30] El Kazdouh H, El-Ammari A, Bouftini S, El Fakir S, El Achhab Y (2018). Adolescents, parents and teachers’ perceptions of risk and protective factors of substance use in Moroccan adolescents: a qualitative study. Subst Abuse Treat Prev Policy.

[31] Rachmatullah A, Ha M, Park J. Relations among education, religiosity and socioeconomic variables. South Afr J Educ. 2019;39(1).

[32] O’Malley PM, Johnston LD, Bachman JG, Schulenberg JE, Kumar R (2006). How substance use differs among American secondary schools. Prev Sci.

[33] Koplewicz HS, Gurian A, Williams K (2009). The era of affluence and its discontents. J Am Acad Child Adolesc Psychiatry.

